# Identification and Characterization of *Eimeria tenella* Microneme Protein (EtMIC8)

**DOI:** 10.1128/spectrum.00228-21

**Published:** 2021-08-25

**Authors:** Ningning Zhao, Shuzhen Ming, Lingyu Sun, Bingxiang Wang, Hongmei Li, Xiao Zhang, Xiaomin Zhao

**Affiliations:** a Department of Preventive Veterinary Medicine, College of Veterinary Medicine, Shandong Agricultural University, Taian City, Shandong Province, China; b Shandong Provincial Key Laboratory of Animal Biotechnology and Disease Control and Prevention, Shandong Agricultural University, Taian City, Shandong Province, China; c Shandong Provincial Engineering Technology Research Center of Animal Disease Control and Prevention, Shandong Agricultural University, Taian City, Shandong Province, China; University of Georgia

**Keywords:** *Eimeria tenella*, EtMIC8, EGF, adhesion, invasion

## Abstract

Microneme proteins (MICs) of Eimeria tenella play key roles in motility, migration, attachment, and invasion processes. More than 20 apicomplexan parasite’s MICs have been identified, with nine *Eimeria* MICs being reported. In this study, a novel E. tenella MIC was identified, and its gene structural features, developmental expression levels, localization, role in adhesion and invasion, and immunogenicity were studied. The results showed that the open reading frame was 1,650 bp, encoding 550 amino acids. It contains a signal sequence, a transmembrane region, four low-complexity boxes, and five epidermal growth factor-like domains (EGF). Subcellular localization revealed its distribution on the membrane surface of the parasite. These characteristics are consistent with the common features of MICs and are named EtMIC8. Anti-EtMIC8 antibodies recognized a specific binding of about 100 kDa in E. tenella, which was twice as large as the prokaryotic expression (about 50 kDa), suggesting that MIC8 may exist naturally as a dimer. EtMIC8 was expressed at higher levels in sporozoites (3.08-fold) and merozoites (2.1-fold) than in sporulated oocysts. The attachment assays using a yeast surface display of MIC8 and its different domains showed that the adherence rates of EtMIC8 to host cells were significantly higher than those of the control (3.17-fold), which was the full contribution of EGF, but neither was alone. Anti-EtMIC8 antibodies significantly reduced the invasion rate of sporozoites into host cells compared to those of the control (*P *< 0.01). Recombinant EtMIC8-EGF peptides could provide moderate protective efficacy (anticoccidial index [ACI]: 169.7), induce humoral responses, and upregulate CD3^+^CD8^+^ lymphocyte cells.

IMPORTANCE

Microneme is a conserved and specialized apical secretory organelles of apicomplexan parasite. Proteins secreted by microneme (MICs) distribute over the surface of parasites when contacted with the host cells and play important roles in parasites’ gliding motility, migration, adhesion, and host cell invasion. Although numerous MICs of the apicomplexan were reported, only nine *E. tenella* MICs have been identified, and more work need to be done. The work reported here newly identifies a microneme protein of *E. tenella*, which plays a role in adhesin and invasion process and has good immunogenicity. These results will provide theoretical support for further understanding of the invasion mechanism of *E. tenella* and provide data support for the development of subunit vaccines.

## INTRODUCTION

Protozoa of the phylum Apicomplexa, such as *Plasmodium*, *Toxoplasma*, and *Eimeria*, are of enormous medical and veterinary importance as pathogens that cause several human and veterinary diseases worldwide (1, 2). These groups are obligate intracellular pathogens whose ability to invade and egress from host cells is essential for their survival and dissemination. A variety of proteins are involved in parasite invasion, intracellular survival, and developmental processes, among which microneme proteins (MICs) play important roles. MICs contain a large array of proteins, which underpin many biological functions such as vacuolar escape, motility, rhoptry secretion, adhesins, and invasion (3–5). Plasmodium berghei perforin-like proteins (PbPLPs) released from microneme compromise the integrity of the parasitophorous vacuole (PV) and host cell membrane for egress (6). Rhoptry protein discharge is dependent on MICs, such as Toxoplasma gondii microneme protein 8 (TgMIC8) and Plasmodium falciparum erythrocyte binding antigen-175 (PfEBA175) (7). The T. gondii cytoplasmic C terminus of several MICs can bind to a glideosome-associated connector (TgGAC) that are linked to the parasite’s actomyosin system, which is critical for gliding motility (7–9). The ability of MICs to bind to the host cell surface through recognition of sialylated oligosaccharides, heparin, glycosaminoglycans, and sialic acid mediates the adhesion process of parasites (10–12). MICs interact with rhoptry neck proteins (RONs) to form a moving junction (MJ) to enter the host cell. In Apicomplexa, a number of MICs have been reported, and many of their functions have been studied. Lal et al. (13) separated and purified *Plasmodium* microneme, and 59 hypothetical proteins with secretory features have been identified (13). Liu et al. (7) summarized the MICs of T. gondii and found that more than 20 MICs have been identified, including TgMIC1-16, TgM2AP, TgAMA1, TgSUB1, TgSPATR, TgROM1, TgTLN4, and TgPLP1 (7). To date, 9 MICs of Eimeria tenella have been reported, including MIC1 to 7 and apical membrane antigen 1 and 2 (AMA1, 2). In the present study, a new EtMIC was identified and characterized.

## RESULTS

### Identification of E. tenella MIC8.

**FIG 1:**
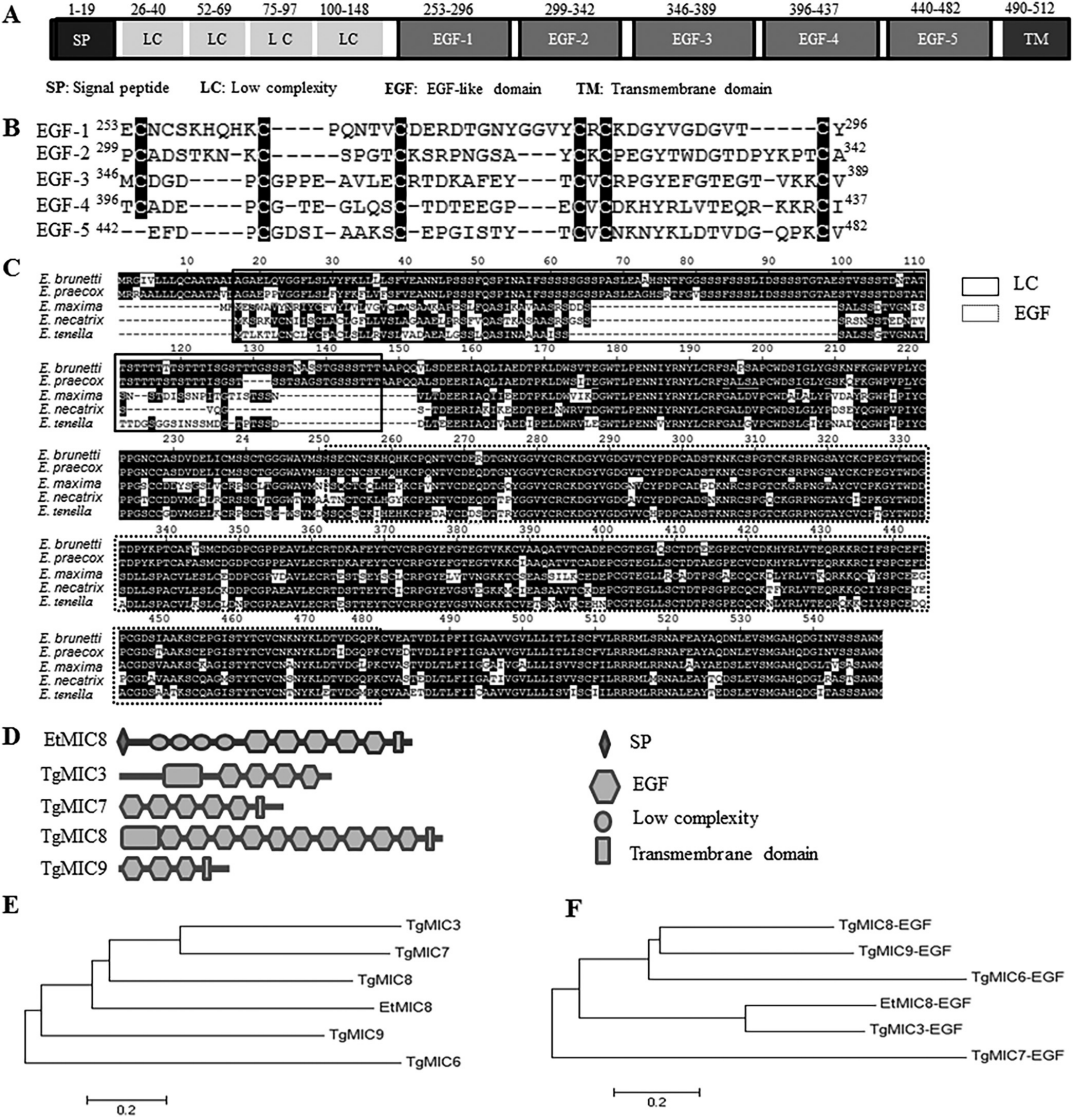
Identification and characterization of EtMIC8. (A) Analysis of the primary structure of EtMIC8. (B) Sequence of the five EGF domains of EtMIC8 with six conserved cysteine residues in the box. (C) Alignment analysis of the MIC8 homologous protein amino acid sequence in different *Eimeria* species that infect chickens. (D) Description of the schematic representations of MICs containing EGF domains from T. gondii. (E) Phylogenetic analysis of the relationship between EtMIC8 and TgMIC3, TgMIC6, TgMIC7, TgMIC8, and TgMIC9. (F) Evolutionary relationship between the EGF domain of EtMIC8 and the EGF domains of TgMIC3, TgMIC6, TgMIC7, TgMIC8, and TgMIC9.

An EtMIC cDNA was cloned from E. tenella strain SD-01. The homology search analysis showed that it was 100% identical to the sequence published in GenBank (XM_013376052.1). The cDNA sequence has a length of 1,650 bp and encodes a peptide of 550 amino acids with a predicted molecular mass of 48.6 kDa. Bioinformatics analysis revealed that it has a signal peptide at the N terminus (1 to 19 amino acids [aa]), followed by low-complexity fragments and four tandemly arranged EGF-like domains (253 to 296 aa, 299 to 342 aa, 346 to 389 aa, 396 to 437 aa) with an incomplete EGF-like domain (440 to 482 aa) and a transmembrane domain at the C terminus (490 to 512 aa) (Fig. 1A). The four EGF-like domains contained six conserved cysteine residues (Fig. 1B). These characteristics are consistent with the MIC family. In accordance with the MICs naming convention, the protein bears the name EtMIC8.

The amino acid sequence of EtMIC8 was used to search for homologous genes of chicken *Eimeria* spp., and a total of four homologous genes were found. Comparative amino acid sequence analysis showed that EtMIC8 had 95.63% identity to Eimeria necatrix (XP_013433095.1), 66.48% identity to Eimeria maxima (XP_013333380.1), 66.49% identity to Eimeria brunetti MIC8 (CDJ53572.1), and 66.3% identity to Eimeria praecox (CDI86929.1) (Fig. 1C). MICs containing the EGF domain of T. gondii were analyzed to identify homologous proteins in closely related species in phylum Apicomplexa. Any TgMICs that have been identified are not homologous to EtMIC8 (Fig. 1D). The evolutionary phylogenetic relationship analysis showed that EtMIC8 was divided into different clusters with TgMIC3, TgMIC7, TgMIC8, and TgMIC9 (Fig. 1E). Structurally, EtMIC8 is similar to TgMIC7 in terms of EGF domain composition but without the signal peptide and low-complexity boxes at the N terminus of TgMIC7. The sequence length of EtMIC8 and TgMIC7 are also different. In addition, the EGF domain of EtMIC8 is highly correlated with that of TgMIC3 as far as evolutionary relationships are concerned (Fig. 1F).

### Localization and dynamic expression of EtMIC8.

**FIG 2:**
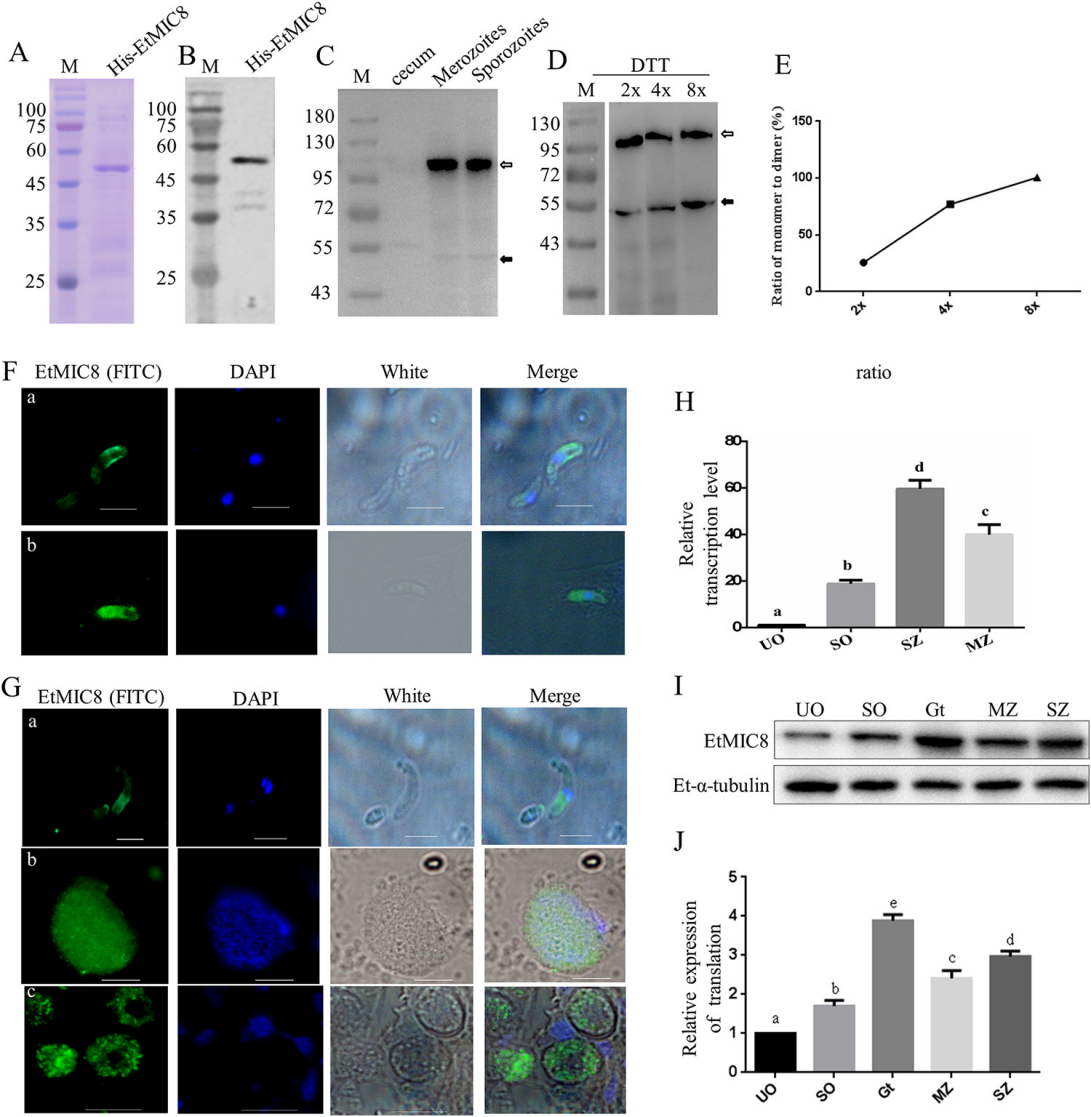
Expression and localization of EtMIC8. (A) Analysis of expression of recombinant His-EtMIC8 in E. coli by SDS-PAGE. (B) Identification of recombinant His-EtMIC8 by Western blotting using anti-His MAb as primary antibody and horseradish peroxidase (HRP)-conjugated goat anti-mouse IgG as secondary antibody. (C) Identification of endogenous expression of EtMIC8 in E. tenella by Western blotting, using anti-EtMIC8 MAb as primary antibody and HRP-conjugated goat anti-mouse IgG as secondary antibody; white arrow refers to the dimer and black arrow refers to the monomer. (D) Merozoites proteins were dissociated with different concentrations of DTT and analyzed by Western blotting, using anti-EtMIC8 MAb as primary antibody. The labels 2×, 4×, and 8× represent 2, 4, and 8 times the normal amount of DTT (100 mM), respectively. (E) Quantification of protein signal in panel D, and the ratio of monomer to dimer was calculated. (F) Localization of EtMIC8 in E. tenella sporozoites. Sporozoites were labeled with anti-EtMIC8 MAb as primary antibody and FITC-conjugated goat anti-mouse secondary antibodies under nonpermeabilization (a) and permeabilization (b) conditions. (G) Identification of EtMIC8 expression in E. tenella merozoites (a), schizonts (b), and gametophytes (c) by IFA, using anti-EtMIC8 MAb as primary antibody. (H) The transcription level of EtMIC8 at different developmental stages in E. tenella. UO, unsporulated oocysts; SO, sporulated oocysts; SZ, sporozoites; MZ, merozoites. Values with different letters are significantly different (*P *< 0.05). (I) EtMIC8 protein levels in E. tenella different developmental stages were detected by Western blotting. Gt, gametophytes. (J) EtMIC8 relative expression levels. Values with different letters are significantly different (*P *< 0.05).

Recombinant EtMIC8 (rEtMIC8) was expressed in Escherichia coli for preparation of anti-EtMIC8 polyclonal and monoclonal antibodies. The E. coli-expressed rEtMIC8 protein was recognized by the anti-His monoclonal antibody with a molecular weight of approximately 50 kDa (Fig. 2A and B). The anti-EtMIC8 polyclonal antibody and two mouse MAbs were prepared and identified. The titers and isotypes of MAb are summarized in Table 1. The native EtMIC8 protein in sporozoites and merozoites of E. tenella was detected by Western blotting using the specific anti-EtMIC8 MAb. The results showed that the anti-EtMIC8 MAb specifically recognized a strong protein band of about 100 kDa (Fig. 2C, white arrow) and a very weak band of about 50 kDa (Fig. 2C, black arrow) that is about the theoretical size of the EtMIC8, suggesting that EtMIC8 may exist in the form of a dimer in E. tenella. Further breaking down test results of the dimer showed that increasing the amount of dithiothreitol (DTT) could reduce dimers and increase monomers (Fig. 2D). Two, four, and eight times the normal amount of DTT made the ratio of monomers to dimers 0.26, 0.77, and 1.00 (Fig. 2E), respectively. The similar result was obtained with β-mercaptoethanol as a reducing agent.

**TABLE 1:** Titers and isotypes of anti-EtMIC8 MAbs

MAb	Titer	Isotype
4-A7	0.81 ± 0.03	IgG1-κ
5-B3	0.68 ± 0.02	IgG1-κ

The results of immunofluorescence assay (IFA) revealed that the EtMIC8 protein was distributed mainly on the surface of the parasite under nonpermeabilization conditions (Fig. 2Fa) and in the cytoplasm and around the surface of the parasites end under permeabilization conditions (Figure 2Fb), confirming that the protein is a membrane protein. The EtMIC8 in merozoites, schizonts, and gametophytes were also immunolabeled by anti-EtMIC8 MAb. Results showed that EtMIC8 were expressed in merozoites (Figure 2Ga), schizonts (Fig. 2Gb), and gametophytes (Figure 2Gc).

The results of detecting transcription levels showed that the mRNA level of EtMIC8 was 18.3-fold higher in sporulated oocysts, 56.4-fold higher in sporozoites, and 38.5-fold higher in merozoites than in unsporulated oocysts (Fig. 2H). The transcription levels of EtMIC8 were significantly higher in sporozoites and merozoites than in sporulated oocysts (*P *< 0.01). The protein levels of EtMIC8 in different developmental stages of E. tenella were examined by Western blotting. Results showed that EtMIC8 expressed in unsporulated oocysts, sporulated oocysts, sporozoites, merozoites, and gametophytes of E. tenella can be recognized significantly by anti-EtMIC8 MAb (Fig. 2I). The EtMIC8 protein expression levels were 2.97-fold higher in sporozoites, 2.4-fold higher in merozoites, and 3.88-fold higher in gametophytes than in unsporulated oocysts (Fig. 2J).

### Adhesion to host cell.

**FIG 3:**
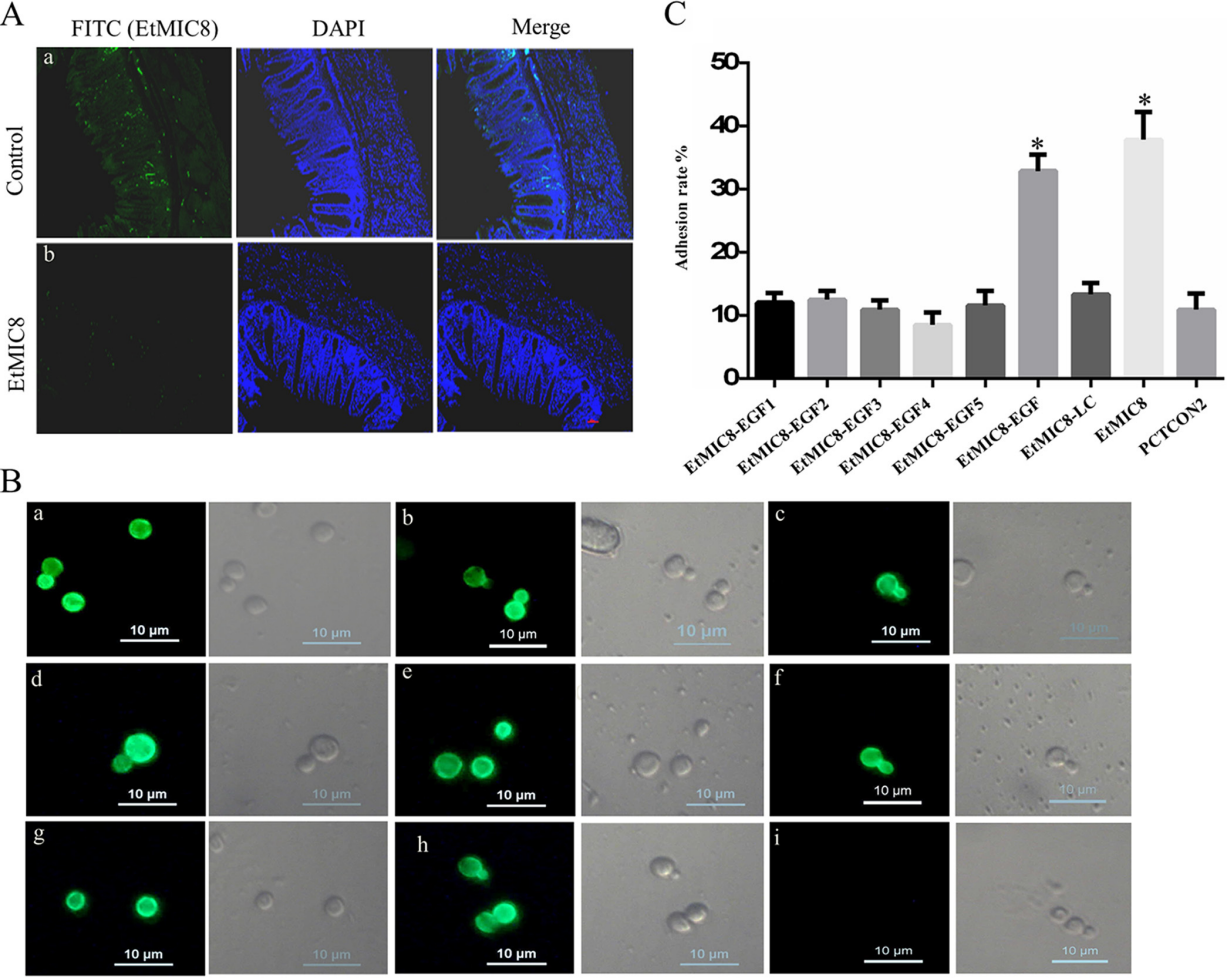
Adhesion assay of EtMIC8 to host cells. (A) Binding of EtMIC8 to cecum of chicken. Tissue sections from cecum were incubated with rEtMIC8 protein. Sections sequentially incubating with anti-EtMIC8 MAb and FITC-conjugated goat anti-mouse IgG and observed by fluorescence microscopy (a). Sections incubated with PBS were used as the controls (b). The binding ability was observed by immunofluorescence assay, and obvious green fluorescence was considered positive. (B) Fluorescence microscopic photographs of yeast cells displaying EtMIC8 (a), EtMIC8-LC (20 to 252 aa) (b), EtMIC8-EGF1 (253 to 296 aa) (c), EtMIC8-EGF2 (299 to 342 aa) (d), EtMIC8-EGF3 (346 to 389 aa) (e), EtMIC8-EGF4 (396 to 437 aa) (f), EtMIC8-EGF5 (440 to 482 aa) (g), and EtMIC8-EGF (253 to 482 aa) (h), using anti-HA MAb as primary antibody and FITC-conjugated goat anti-mouse IgG as secondary antibody. Yeast cells displaying EtMIC8 were labeled without primary antibody as a control. (C) Adhesion rate of yeast cells displayed EtMIC8 and its different domains to DF1 cells. Yeast transfected with pCTCON2 plasmid as control. The assays were performed in triplicate. *, *P* < 0.05 represents the difference compared to the control.

To explore the role of EtMIC8 in the binding ability of host cell, the binding ability of EtMIC8 to cecum of chickens was performed as describe in Materials and Methods. The results showed that the binding of EtMIC8 to cecum was clearly labeled by IFA using anti-EtMIC8 MAb in rEtMIC8 protein incubation group (Fig. 3Aa), and no fluorescence was observed in the control (Fig. 3Ab), suggesting that EtMIC8 has the ability to bind to cecum of chickens.

In order to detect whether EtMIC8 or its different domains play a key role in host cell adhesion using yeast surface display adhesion model, the different yeast strains displaying EtMIC8 or its different domains were constructed. The results showed that the peptides of EtMIC8 (Fig. 3Ba), EtMIC8-LC (20 to 252 aa) (Fig. 3Bb), EtMIC8-EGF1 (253 to 296 aa) (Fig. 3Bc), EtMIC8-EGF2 (299 to 342 aa) (Fig. 3Bd), EtMIC8-EGF3 (346 to 389 aa) (Fig. 3Be), EtMIC8-EGF4 (396 to 437 aa) (Fig. 3Bf), EtMIC8-EGF5 (440 to 482 aa) (Fig. 3Bg), and EtMIC8-EGF (253 to 482 aa) (Figure 3Bh) were expressed on the surfaces of different yeast strains revealed by IFA labeling assays using specific MAbs, either anti-hemagglutinin (anti-HA; attached to C terminus, for domains) or anti-EtMIC8, which indicated that the different yeast strains displaying EtMIC8 or different domains were built up successfully. The different yeast strains displaying EtMIC8 or its different domains were used to test their respective adhesion abilities to DF-1 cells *in vitro*. The results showed that the adhesion rates of the yeast groups displaying EtMIC8 and EtMIC8-EGF (253 to 482 aa) were 36.2% and 32.1%, respectively, which were significantly higher than that of the control group transformed with empty vector (10.4%) (*P* < 0.05) (Fig. 3C). The adhesion rates of the yeast groups displaying EtMIC8-LC, EtMIC8-EGF1, EtMIC8-EGF2, EtMIC8-EGF3, EtMIC8-EGF4, and EtMIC8-EGF5 were 13.3%, 12.2%, 12.5%, 10.9%, 9.1%, and 11.6% respectively, which were not significantly different from that of the control group (*P *> 0.05) (Fig. 3C). These results suggest that the adhesion ability of EtMIC8 to host cells is a contribution of five EGF domains working together, rather than that of any one of them.

### Invasion ability of EtMIC8 to host cells.

**FIG 4:**
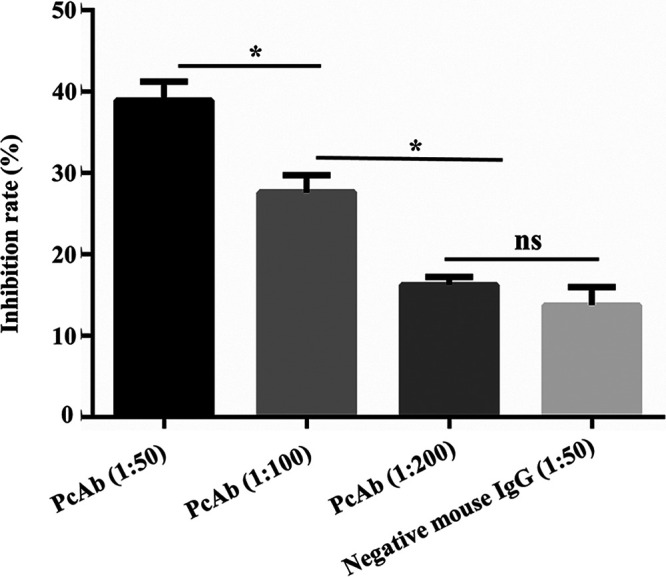
Inhibition of sporozoite invasion by anti-EtMIC8 antibody *in vitro*. Invasion-inhibitory activities of anti-EtMIC8 antibody at different dilutions. The assays were performed in triplicate. Negative mouse IgG diluted at 1:50 was performed as control. *, *P* < 0.05 represents the difference compared to the control.

The results of the polyclonal antibody inhibition test showed that pretreatment with anti-EtMIC8 sera effectively reduced the ability of sporozoites to invade host cells in a dose-dependent manner (*P *< 0.05). EtMIC8-specific polyclonal antibodies blocked 38.25% ± 5.32% of sporozoites from invading DF-1 cells at a concentration of 50-fold dilution and 27.64% ± 5.32% at 100-fold dilution, which were significantly higher than that of the mouse serum control group at a concentration of 50-fold dilution (13.23% ± 2.29%) (Fig. 4). The results suggest that EtMIC8 may be involved in the sporozoite invasion process.

### Protective efficacy of recombinant EGF domains (rEtMIC8-EGF).

The protective efficacy of the rEtMIC8-EGF peptide against E. tenella was evaluated based on anticoccidial index (ACI), including body weight gain, cecal lesion score, and oocyst output. No dead chickens were found in any group after exposure to E. tenella. Body weight gain, lesion scores, oocyst shedding, and ACI data of every group are described in Table 2. The body weight gain of chickens in the rEtMIC8-EGF peptide group was significantly higher than that in the unimmunized challenged group (PBS-II group) and not significantly different compared to that in the unimmunized unchallenged group (PBS-I group). Chickens immunized with EtMIC8-EGF protein displayed lesion scores and oocyst shedding significantly lower than those of the PBS-II group. These results indicate that EtMIC8-EGF has the capability to moderately resist the activity of 8,000 E. tenella sporulated oocyst infecttions with an ACI as high as 168.7.

**TABLE 2:** Protective effect of the recombinant EtMIC8-EGF peptide against oocysts of E. tenella infection in chicken^*a*^

Group	Avg body wt gain (g)	Oocyst shedding (×10^6^/g)	Lesion score	ACI
EtMIC8-EGF	110.94 ± 3.464^cb^	2.62 ± 0.206^*a*^	1.33 ± 0.211^*a*^	168.7
PBS-II	94.43 ± 2.845^*a*^	5.04 ± 0.337^b^	2.17 ± 0.307^b^	118.4
PBS-I	117.9 ± 2.669^b^	0	0	200

a PBS-I group was immunized by PBS without E. tenella challenge; PBS-II group was immunized by PBS and challenged with E. tenella. Values with different letters in same column are significantly different (*P* < 0.05). Excellent activity: ACI > 180; moderate activity: 179 > ACI  > 160; limited activity: 159 > ACI > 120; nonactivity: ACI < 120.

### Capability of EtMIC8-EGF to induce humoral and cellular immunity.

**FIG 5:**
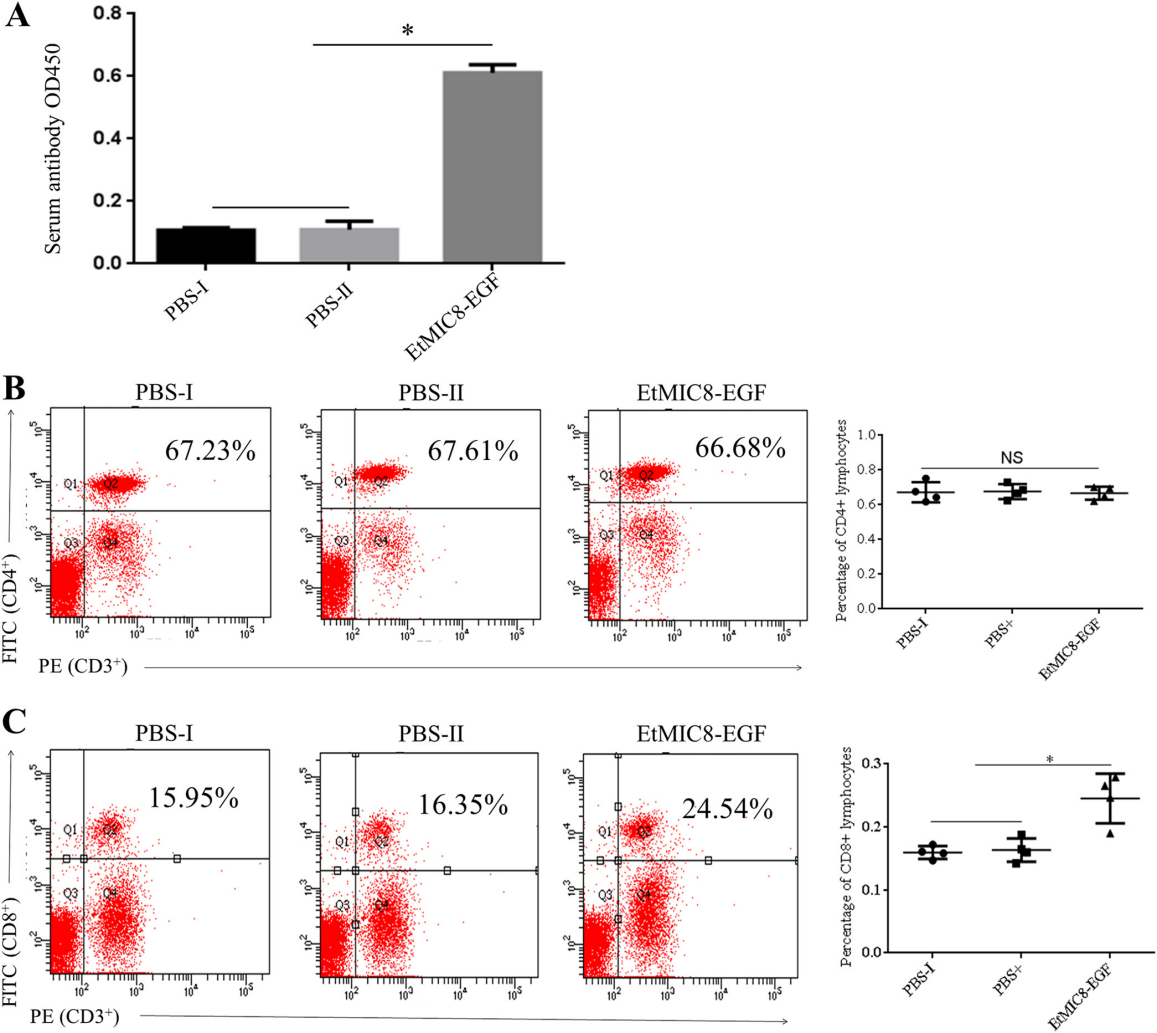
Humoral and cellular immune responses induced by the rEtMIC8-EGF peptide. (A) Serum IgG antibody levels against rEtMIC8-EGF peptide measured at day 7 after the second immunization. The percentages of CD3^+^CD4^+^ (B) and CD3^+^CD8^+^ (C) in the blood on day 7 after the second immunization. *, *P* < 0.05 represents significant difference.

To evaluate the humoral and cell-mediated immune responses stimulated by the recombinant EtMIC8-EGF protein, serum antibody levels and blood lymphocyte subpopulations were measured. At 14 days postimmunization, the serum antibody level in the EtMIC8-EGF group was significantly higher than that in chickens immunized with phosphate-buffered saline (PBS; PBS-I and PBS-II) (Fig. 5A). There was no significant difference in the percentage of peripheral blood CD3^+^CD4^+^ lymphocyte cells between the EtMIC8-EGF and PBS groups (Fig. 5B). However, the CD3^+^CD8^+^ lymphocyte cells were significantly higher in the EtMIC8-EGF immune group than in the control group (Fig. 5C). These results suggest that EtMIC8-EGF both induces humoral immunity and upregulates cellular immunity, which is mainly manifested by the activation of CD3^+^CD8^+^ lymphocyte cells.

## DISCUSSION

Microneme is a conserved and specialized apical secretory organelle of apicomplexan parasites, which secrete proteins during the invasive phase of the parasite (7, 14). As soon as the parasites come into contact with their host cells, MICs are distributed on the surface of the parasites and are involved in parasite-host cell interactions (15, 16). The sequence and structure of MICs also differ considerably in different apicomplexan parasites due to differences in the parasitic environment and host cells (17, 18). To perform similar biological functions in host cells, including adhesion, invasion, and survival, different MICs usually retain some common features. Most MICs are secretory or membrane proteins containing a limited number of copies of adhesive domain types, such as epidermal growth factor (EGF) domains, apple domain of von Willebrand factor, thrombospondin-related anonymous protein (TRAP), and chitin binding-like (CBL) (3, 7). These adhesive domains are believed to be characteristic of apicomplexan MICs, which allow the identification of a large number of additional putative MICs bearing these domains in the parasite database (19, 20). In the present study, a novel E. tenella MIC containing five tandem EGF domains was identified. Specific antibodies were clearly labeled with EtMIC8 on the surface of the parasite, implying that EtMIC8 is a surface membrane-associated protein. These features match those common to the microneme group of proteins, which suggests that EtMIC8 is a novel E. tenella MIC. Compared to that between *E. maxima* (66.49% identified), *E. brunetti* (66.49% identified), and *E. praecox* (66.3% identified), the homology between EtMIC8 and *E. necatrix* was 95.63%, which may be related to the common parasitic characteristic of E. tenella and *E. necatrix* in the cecum.

Adhesion and invasion processes are key steps for apicomplexan parasites infection (21). These processes are mediated by a variety of parasite proteins that interact with host cells (22–24). Studies have shown that MICs play an important role in the adhesion and invasion of parasites to host cells due to their multiple adhesion domains. TgMIC1 and TgMIC13 could bind to sialic acid on the gut epithelial cell wall, thereby preventing the excretion of sporozoites from the intestine. MICs containing thrombospondin-1 (TSP-1) mediate parasite binding to host ligands (7, 17). The adhesion domain of TgMIC3 and TgMIC8, similar to that of chitin-binding-like (CBL), is involved in the cross-linking of chitin subunits that mediate parasite-cell surface interactions (25). The microneme protein AMA1 is capable of forming a ring-like structure that mediates T. gondii invasion of host cells (26). EGF is an evolutionarily conserved protein domain found in many apicomplexan MICs (27–29). It typically contains about 45 residues and 3 disulfide bonds, of which a considerable portion seem to be mediated mainly by protein-protein interactions. Several studies have shown that the EGF domains of these proteins are involved in the adhesion and invasion of host cells. Both TgMIC6 and NcMIC6 have the ability to adhere to host cell ligands (7, 30), and the second and third EGF domains promote host cell receptor recognition (31). Plasmodium vivax merozoite surface protein 1 (PvMSP1) was considered a mediator of P. vivax adherence to reticulocytes, and the EGF domain played a key role in mediating this process (23). In the present study, adhesion assays both *in situ* and *in vitro* demonstrated the ability of EtMIC8 to adhere to cecum of chickens and DF-1 cells. This adhesion function requires the simultaneous existence of five EGF domains. The results of our sporozoite invasion assay showed that specific anti-EtMIC8 antibodies significantly inhibited the invasive ability of sporozoites. These results suggest that EtMIC8 may be involved in the attachment and invasion of host cells, which is supported by the results of significantly higher transcript and expression levels in sporozoites and merozoites.

Many proteins perform biological functions as complexes. MICs often form heterologous complexes with other proteins in Apicomplexa protozoa (7). TgMIC1/MIC4/MIC6 is a complex which was first identified in T. gondii (32). TgMIC2 remains associated with TgM2AP during the invasion process (33). TgAMA1 forms moving junctions along with TgRON2, TgRON4, TgRON5, and TgRON8, which facilitates propulsion of the parasite into the host cell (34). Dimerization is a common phenomenon that plays a key role in protein function, such as increasing protein stability, signal transduction, and ligand binding (35, 36). TgMIC3 is reported to be a dimeric 90-kDa protein, synthesized as a 40-kDa precursor that is proteolytically processed to a 38-kDa end product during its trafficking via the secretory pathway and prior to storage in mature organelles. Dimerization is essential for the adhesion of TgMIC3 to the host cell surface (27). In the present study, native EtMIC8 was detected by specific anti-EtMIC8 MAb in E. tenella sporozoites and merozoites in a denaturing gel (100 mM DTT as a reducing agent). A strong band of about 100 kDa and a very weak band of about 50 kDa that was about the theoretical size of the EtMIC8 were recognized by specific anti-EtMIC8 MAb. This suggests that EtMIC8 may function as a dimer in E. tenella. Increasing the amount of DTT raised the monomer amount and decreased the dimer amount, suggesting that the dimers may be formed by disulfide bonds. Up to 8 times the amount of normal DTT still could not fully open the dimers, indicating that the dimerization is very strong. This may not be surprising in consideration of the EtMIC8 harboring 40 cysteines. Furthermore, dimerization of proteins may be involved in different mechanisms, such as genetic domain fusion, domain swapping, or a gene encoding a dimerization domain like a coiled-coil domain, and formed through noncovalent bonds, such as hydrogen bonds, ionic bonds, van der Waals interactions, hydrophobic bonds, and covalent bonds, like disulfide bonds, in different biology systems (37–39). It is possible that the EtMIC8 complex might be involved in multiple mechanisms that are worthy of further study.

Studies have shown that EGF domains appear to play a central role in immune responses. A recombinant protein containing the EGF domains from Plasmodium yoelii merozoite surface protein-1 (PvMSP-1) was used as a subunit vaccine to immunize mice, thereby protecting the immunized mice against a lethal attack by the same parasite strain (40). The EGF domain of TgMIC3 has been reported to induce a strong cellular immune response (41). Numerous *Plasmodium* merozoite surface proteins (PMSPs) containing the EGF domains have been considered good candidate vaccine antigens (41, 42). In the present study, our results also revealed that the EGF domains of EtMIC8 showed higher immunogenicity and protective efficacy, with an ACI as high as 169.7. It is widely accepted that cell-mediated immune responses, particularly those associated with CD8^+^ T lymphocytes, are the main mediators of immunity against E. tenella infection (43). CD8^+^ cells are well known for their ability to simultaneously produce high levels of interferon gamma (IFN-γ) and interleukin 10 (IL-10) in response to parasites (44). Studies have shown that TgMIC antigens, such as MIC1, MIC3, MIC4, and MIC6, have the ability to induce memory T cell recall responses, thereby maintaining CD8^+^ T cells at high levels (45). The EGF domain of P. yoelii merozoite surface protein-1 (MSP-1) can reduce pathogen infection by inducing a CD8-mediated cellular immune response (41). The results of the immune experiment suggest that the EGF domains of EtMIC8 peptide have the ability to induce CD3^+^CD8^+^ cell differentiation, which may be beneficial for the clearance of pathogens.

In conclusion, a novel EtMIC, named EtMIC8, has been cloned and identified. It may exist as a dimer in E. tenella and plays a role in the adhesion and invasion of host cells, with good immunogenicity.

## MATERIALS AND METHODS

### Ethical statement.

The study protocol and all associated animal studies were approved by the Animal Care and Use Committee of Shandong Agricultural University, Tai’an, China (approval number: SDAUA-2019-018).

### Strains, parasites, antibody, and experimental animals.

Saccharomyces cerevisiae EYB100 strain stored in our laboratory was used for yeast surface display. The wild-type E. tenella strain SD-01 was isolated and stored in our laboratory (46). Anti-His MAb and anti-HA MAb were purchased from Solarbio (Beijing Solarbio Science & Technology Co., Ltd.). The anti-EtMIC8 MAb 4-A7 was prepared and stored in our laboratory. The chicken embryo fibroblast cell line (DF1) cultured in Dulbecco’s modified Eagle medium (DMEM; Gibco) with 10% fetal bovine serum (FBS) in a 5% CO_2_ cell incubator at 37°C was used for the inhibition assay. One-day-old Hy-Line Brown cocks were purchased from the hatchery of Hailan (Tai’an, China) and raised under coccidia-free conditions.

### Preparation of different developmental stages of E. tenella.

E. tenella unsporulated oocysts were obtained from the cecal contents of chickens infected orally with 1 × 10^4^ sporulated oocyst at 7 days postinfection and were purified by saturated sodium chloride flotation method (47). Sporulated oocysts were obtained from incubation of unsporulated oocysts in 2.5% potassium dichromate solution at room temperature for 2 to 3 days. Sporozoites were obtained from sporulated oocysts as described in reference 48. Briefly, sporulated oocysts were sterilized in 20% sodium hypochlorite for 10 min at room temperature and broken down by grinding with glass beads to release sporocysts. The sporozoites were excysted by incubating with 0.25% trypsin (Beijing Solarbio Science & Technology Co., Ltd.) and 0.25% sodium taurocholic acid in sterile PBS at 41°C for 40 to 60 min. The free sporozoites were collected by filtering through a G3 funnel. Merozoites were isolated at 120 h from ceca of chicken infected orally with 1 × 10^4^ sporulated oocyst (49). The ceca were incubated with hyaluronidase (0.5 mg/ml) in PBS at 37°C for 30 min to release merozoites. The crude merozoite solution was collected by filtering through four layers of gauze and incubated with erythrocyte disruption solution (Beijing Solarbio Science & Technology Co., Ltd.) for lysing chicken red blood cells. The merozoites were resuspended in 30% Percoll in PBS (P-PBS). Five volumes of merozoites solution were layered gently onto one volume of 50% P-PBS and centrifuged at 2,200 × *g* for 15 min. Merozoites that existed in the lower aqueous layer were collected. Gametophytes were isolated from infected chicken ceca at 134 to 144 h postinfection (50) and purified by Percoll following the merozoite purified method.

### RNA extraction, cDNA synthesis, and gene cloning.

Total RNA was extracted from the second-generation merozoites of E. tenella using TRIzol reagent (TaKaRa, Dalian, China) in accordance with the manufacturer’s protocol. The RNA was treated with DNase I and reverse transcribed into cDNA using reverse transcriptase (TaKaRa, Dalian, China) and oligo (dT) primers. The primers EtMIC8-F and EtMIC8-R (Table 3) were used to amplify the full length of the EtMIC8 gene-carrying region from cDNA of E. tenella.

**TABLE 3:** Primers used in this study

Primer	Sequence (5′–3′)
EtMIC8-F	ATGAGGGGGATTGTTTTGCTGC
EtMIC8-R	CTACATCCACGCGCTGGAACT
PET30a-EtMIC8-F	CCCTCGAGGAACTACAGGTTGGAGGGT
PET30a-EtMIC8-R	GGCTCGAGCTACATCCACGCGCTGGAAC
PET30a-EtMIC8-EGF-F	CGGAGCTCTGTAATTGCAGCAAACACCAAC
PET30a-EtMIC8-EGF-R	CCCTCGAGCACACACTTCGGCTGGCCGTC
pCTCON2-mic8-F	CAGGTCGACTGCGGGGAACTACAGGTTGGAGGGT
pCTCON2-mic8-R	GTCATCCTTGTAATCCTTATCGTCGTCATCCTTGTAATCCTACATCCACGCGCTGGAAC
pCTCON2-mic8-EGF-1-F	CAGGTCGACTGCGGGGAATGTAATTGCAGC
pCTCON2-mic8-EGF-1-R	GTCATCCTTGTAATCCTTATCGTCGTCATCCTTGTAATCGTAGCAGGTGACCCCGTC
pCTCON2-mic8-EGF-2-F	CAGGTCGACTGCGGGTAAGCCCTGTGCGGACTCCAC
pCTCON2-mic8-EGF-2-R	GTCATCCTTGTAATCCTTATCGTCGTCATCCTTGTAATCCGCGCATGTGGGTTTGTAAG
pCTCON2-mic8-EGF-3-F	CAGGTCGACTGCGGGATGTGCGACGGGGATCC
pCTCON2-mic8-EGF-3-R	GTCATCCTTGTAATCCTTATCGTCGTCATCCTTGTAATCGACACACTTCTTTACCGTG
pCTCON2-mic8-EGF-4-F	CAGGTCGACTGCGGGTGCGCCGACGAACCCTG
pCTCON2-mic8-EGF-4-R	GTCATCCTTGTAATCCTTATCGTCGTCATCCTTGTAATCGATGCACCGTTTTTTCCG
pCTCON2-mic8-EGF-5-F	CAGGTCGACTGCGGGGAATTCGATCCCTGTGG
pCTCON2-mic8-EGF-5-R	GTCATCCTTGTAATCCTTATCGTCGTCATCCTTGTAATCCACACACTTCGGCTGGCC
pCTCON2-mic8-Low-F	CAGGTCGACTGCGGGGAACTACAGGTTGGAGG
pCTCON2-mic8-Low-R	GTCATCCTTGTAATCCTTATCGTCGTCATCCTTGTAATCCGAGGAACTCATGACGGC
RT-EtMIC8-F	TGCGCGTTTGTTAGCATGTG
RT-EtMIC8-R	ACACATTCCGGGCCTTCTTC
RT-18s-F	TGTAGTGGAGTCTTGGTGATTC
RT-18s-R	CCTGCTGCCTTCCTTAGATG

### Bioinformatics analysis.

The online server ExPASY was used to predict the physicochemical properties of EtMIC8, including protein molecular weight, amino acid composition, and isoelectric point. SignalP3.0 (http://www.cbs.dtu.dk/services/SignalP-3.0/) and TMHMM (http://www.cbs.dtu.dk/services/TMHMM/) were used to predict the signal peptide and transmembrane domain. Smart (http://smart.embl-heidelberg.de) was used to identify protein domains in EtMIC8.

### Antibodies preparations.

Anti-EtMIC8 polyclonal antibodies were generated following a previously described method (51). Briefly, five 5-week-old female BALB/c mice were immunized subcutaneously with purified rEtMIC8 (50 μg/mouse) three times at 2-week intervals. Eight days after the final immunization, the serum was tested and separated from the immunized mice. Anti-EtMIC8 MAbs were generated following a previously described method (52). Briefly, mice were immunized subcutaneously with purified rEtMIC8 (50 μg/mouse) 4 times. Four days after the last immunization, spleen cells of mice were isolated and fused with SP20 myeloma cells using standard techniques. The immunoglobulin subclass was determined using IsoStrip mouse monoclonal antibody isotyping kit (Sigma-Aldrich).

### Western blotting.

In order to extract protein, unsporulated oocysts and sporulated oocysts were quickly frozen with liquid nitrogen, and the frozen oocysts were broken by manual grinding with a mortar and pestle. During grinding, liquid nitrogen was added to keep the oocysts in a frozen state until more than 90% of the oocyst walls were broken as observed through a microscope. The broken oocyst, purified sporozoites, merozoites, and gametophytes were lysed in radioimmunoprecipitation assay (RIPA) buffer containing a protease inhibitor cocktail (Beyotime Co., LTD., Beijing, China) on ice for 1 h. Proteins were extracted from lysates by centrifugation at 10,000 × *g* for 30 min and were boiled in denaturing sample buffer (50 mM Tris [pH 6.8], 2% SDS, 1% bromophenol, 10% glycerol, 100 mM DTT). Samples were first resolved by sodium dodecyl sulfate-polyacrylamide gel electrophoresis (SDS-PAGE) and then transferred onto polyvinylidene difluoride (PVDF) membranes (Millipore). After blocking with 5% BSA in Tris-buffered saline and polysorbate 20 (TBST), the PVDF membranes were reacted with primary antibodies and subsequently with the corresponding secondary antibodies conjugated with horseradish peroxidase (HRP). The information on primary and secondary antibodies is listed in Table 4. In order to dissociate the dimer, E. tenella protein was treated with 2, 4, and 8 times the normal amount of DTT (100 mM) at 4°C for 1 h, respectively. The proteins were analyzed by Western blotting, using anti-EtMIC8 MAb.

**TABLE 4:** Information for MAb

MAb	Dilution ratio	Usage	Source
Anti-His MAb (mouse)	1:5,000	Western blotting	Solarbio
Anti-HA MAb (mouse)	1:5,000	Western blotting	Solarbio
	1:100	IFA	
Anti-EtMIC8 MAb 4-A7 (mouse)	1:2,000	Western blotting	Prepared in our lab
	1:50	IFA	
FITC-conjugated goat anti-mouse IgG	1:200	IFA	Solarbio
Horseradish peroxidase (HRP)-conjugated goat anti-mouse IgG	1:10,000	Western blotting	Proteintech
Anti-actin MAb (mouse)	1:5,000	Western blotting	Proteintech
Anti-E. tenella α-tubulin polyclonal antibody	1:1,000		Prepared in our lab

### Immunofluorescence assay.

IFA was used to determine the localization of EtMIC8 and was performed as described previously (53). Briefly, fresh sporozoites, merozoites, schizonts, and gametophytes were fixed, permeabilized, and blocked. The samples were incubated with anti-EtMIC8 MAb 4-A7 as a primary antibody and FITC-conjugated goat anti-mouse IgG as a secondary antibody. After being washed with PBS, the cells were examined by fluorescence microscopy (Nikon-ECLIPSE; Japan).

### Quantitative real-time PCR.

Total RNA was extracted from the four stages of E. tenella using TRIzol reagent (TaKaRa, China) according to the manufacturer’s protocol. cDNA was generated by SuperScriptII reverse transcriptase (TaKaRa, China) using random primers. Quantitative real-time PCR (qRT-PCR) was performed on an ABI 7500 using the SYBR1 green I dye method. A fragment encoding the 18S rRNA of E. tenella was used as the reference gene. Each reaction was carried out in triplicate.

### Binding of EtMIC8 to cecum.

Binding assay was performed as described previously (54). Briefly, cecum tissue samples from 2-week-old chickens without coccidian infection were dehydrated, waxed, and fixed in 4% paraformaldehyde for preparation of tissue sections. Tissue sections were boiled (about 10 min) in sodium citrate buffer (Beijing Solarbio Science & Technology Co., Ltd.) to repair the antigen. The sections were blocked in 5% bovine serum albumin in TBST for 2 h. After cooling to room temperature, sections were incubated with rEtMIC8 protein overnight at 4°C. Samples incubated with PBS were used as the controls. Subsequently, sections were sequentially incubated with anti-EtMIC8 MAb for 1 h at 37°C, the FITC-conjugated secondary antibody for 30 min at 37°C, and DAPI (4′,6-diamidino-2-phenylindole; Beijing Solarbio Science & Technology Co., Ltd.) for 5 min. The tissue sections were detected by fluorescence microscopy (Nikon-ECLIPSE; Japan). The EtMIC8 binding ability that was proportional to the fluorescence intensity was measured by detecting fluorescence intensities of different groups.

### Adhesion assay to DF-1 cell.

The adhesion ability of EtMIC8 or its domains was tested using a yeast surface display adhesion model according to the method described previously (51). Briefly, the EtMIC8 or its domains were expressed transiently on the surface of yeast cells. The yeast cells displaying the EtMIC8 or domains on their surfaces would have an adhesion rate to host cell higher than that of control yeast cells (not displaying the EtMIC8 or domains) if the proteins tested had the adhesion ability. The actual adhesion rate of each group was determined by comparing the adhesion rates between the yeasts displaying the EtMIC8 or domains on their surfaces and the control yeasts. The low-complexity domain (EtMIC8-LC, 20 to 252 aa) and EGF domains (EtMIC8-EGF1, 253 to 296 aa, EtMIC8-EGF2, 299 to 342 aa, EtMIC8-EGF3, 346 to 389 aa, EtMIC8-EGF4, 396 to 437 aa, EtMIC8-EGF5, 440 to 482 aa, and EtMIC8-EGF, 253 to 482 aa) of EtMIC8 and EtMIC8 full sequence were amplified, respectively, by PCR using specific primers (Table 3). The PCR products of EtMIC8 and different domains were cloned, respectively, into pCTCON2 plasmids (a courtesy gift from Dane Wittrup, MIT, USA) using a homologous recombination kit (Vazyme Biotech Co., Ltd.). The yeast strains transformed with pCTCON2 plasmids carrying different EtMIC8 fragments were grown in selective plates (synthetic defined medium with casamino acids [SDCAA]: 0.67% yeast extract, 2% glucose, 0.5% casein acids hydrolysate, 0.15% agar power, 10% ampicillin). The positive strains were confirmed by PCR using specific primers (Table 3) and cultured in the synthetic galactose medium with casamino acid (SG-CAA) (0.67% yeast nitrogen base without amino acids and ammonium sulfate, 2% galactose, 0.5% casein acids hydrolysate) at 30°C for 24 h for expression of EtMIC8 or its domains. The displaying of EtMIC8 or its domains on surfaces of specific yeast strain was observed with a fluorescence microscope through IFA using anti-HA (for domains) or anti-EtMIC8 (for full EtMIC8) MAbs. For adhesion experiments, eight experimental groups (each yeast strain displaying one EtMIC8 fragment was one group) and one control group (yeast strain transformed with empty pCTCON2 vector) were set up. The DF-1 cells were grown to full confluence. Then, 100 yeast cells of each group were added into one well of 24-well plates with cultured DF-1 cells. Each group was repeated in 3 wells and the whole experiment was repeated 3 times. After 2 h of culturation at 30°C, the DF-1 cells were washed three times in PBS. Then, 500 μl SDCAA with 0.8% agar was added to each well of the plates and cultured at 30°C for 36 h. Yeast colonies were counted for each group, and adhesion rates of each group were calculated as follows: adhesion rate = (number of colonies/100) × 100%.

### Sporozoite invasion inhibition assay.

The invasion inhibition assay is based on the observation that E. tenella sporozoites invade DF-1 cells and follows the method described by Wang et al. (28, 51). The sporozoites were preincubated at 37°C with different dilutions of purified antibodies, respectively. DF-1 cells were infected with 2 × 10^5^ sporozoites. After 2 h of culture, the free sporozoites were collected and counted. The percentage of uninfected sporozoites was used to calculate the inhibition rates.

### Evaluation of protective efficacy.

To determine the immunogenicity of adhesion domains, EtMIC8-EGF fragment (253 to 482 aa) was expressed in E. coli in routine molecular biology methods. Ninety chickens were divided randomly into 3 groups (30/group). Chickens were immunized subcutaneously with 50 μg/chicken purified rEtMIC8-EGF peptide in Freund’s complete adjuvant (FCA; Sigma) (group EtMIC8-EGF) or PBS in FCA (PBS-I and II) at 7 days of age. One week after primary immunization, chickens were administered a booster immunization with the same dose of proteins or PBS emulsified with Freund’s incomplete adjuvant (FIA; Sigma), respectively. Seven days after booster immunization, chickens, except those in the PBS-I group, were infected with 8,000 E. tenella sporulated oocysts by oral gavages using gastric tubes. The protective efficacy was evaluated based on the survival rate, body weight gain, oocyst count per gram of feces, cecal lesion score, and ACI, following the method described by Zhao et al. (53). Briefly, body weight gains were determined as the difference in the body weights of chickens between 10 days postinfection (dpi) and after the second immunization. Total feces of each group from 7 dpi to 9 dpi was collected, mixed, and weighted. The oocysts of each group’s fecal sample were isolated and counted using a McMaster counting chamber. The average number of 3 repeats from each group was used to calculate the oocyst numbers of every gram of feces. The cecal lesion scores of the chickens from each group were determined using a 4-grade system (from score 0: no cecal lesions to score 4: severe lesions) at 6 dpi as described previously (55). The ACI of each group was calculated using the following formula: ACI = (survival rate + relative weight gain) − (lesion score + oocyst index).

### Serum antibody levels and blood lymphocyte subpopulation assessment.

At 0 and 5 dpi, the serum antibodies of chickens were detected by enzyme-linked immunosorbent assay (ELISA) as described previously (56). All samples were analyzed in triplicate.

To measure the percentages of CD4^+^ and CD8^+^ cells among CD3^+^ cells, peripheral blood lymphocytes (PBLs) were incubated with phycoerythrin (PE)-conjugated mouse anti-chicken CD3 antibody (Southern Biotech, USA) and fluorescein isothiocyanate (FITC)-conjugated mouse anti-chicken CD4 antibody (Southern Biotech) or FITC-conjugated mouse anti-chicken CD8α antibody (Southern Biotech) at 4°C for 1 h. After being washed with PBS, the PBLs were analyzed by fluorescence-activated cell sorter (FACS; FACSC 2000, BD).

### Statistical analyses.

Graphs were created, and statistical analyses were performed, using GraphPad Prism statistical software package for Windows, version 6.0 (GraphPad software, Inc., La Jolla, USA). Graphs represent means, and error bars represent standard errors of means. All data were analyzed using *t* test. A *P* value of <0.05 was considered indicative of statistically significant differences.
